# An Attenuated *Escherichia coli* K88ac *LT(S63K)*Δ*STb* Efficiently Provides Protection Against Enterotoxigenic *Escherichia coli* in the Mouse Model

**DOI:** 10.3389/fvets.2020.620255

**Published:** 2021-02-12

**Authors:** Xinyu Zhang, Shupei Yu, Darong Cheng, Yu Feng, Yuefei Yang, Huaichang Sun, Jiabo Ding, Fang Wang

**Affiliations:** ^1^College of Veterinary Medicine, Yangzhou University, Yangzhou, China; ^2^Jiangsu Co-innovation Center for Prevention and Control of Important Animal Infectious Diseases and Zoonoses, Yangzhou, China; ^3^Department of Biologics Detection Technology, China Institute of Veterinary Drugs Control, Beijing, China

**Keywords:** ETEC, K88ac, LT(S63K), STb deletion, attenuation, protection

## Abstract

To develop an attenuated vaccine candidate against K88ac enterotoxigenic *Escherichia coli* (ETEC), a novel *Escherichia coli* (*E. coli*) K88ac *LT(S63K)*Δ*STb* with *LT(S63K)* mutation and *ST1* deletion was generated using site mutagenesis and λ-Red homologous recombination based on wild paternal ETEC strain C83902. *E. coli* K88ac *LT(S63K)*Δ*STb* showed very similar fimbriae expression and growth kinetics to the wild strain C83902, but it was significantly attenuated according to the results of a rabbit ligated ileal loop assay and mouse infection study. Oral inoculation with *E. coli* K88ac *LT(S63K)*Δ*STb* stimulated the mucosa immune response and induced the secretion of IgA to K88ac in the intestines in mice. A challenge experiment revealed that the attenuated strain provided efficient protection against C83902 in the following 7 days and at the 24th day post-inoculation, suggesting that the attenuated isolate could act as an ecological protectant and vaccine in preventing K88ac ETEC.

## Introduction

Porcine neonatal diarrhea is a global problem in the pig industry (1). It is mainly caused by enterotoxigenic *Escherichia coli* (ETEC) strains, which may express fimbriae K88 (F4) or F18 related to bacterial colonization in the intestine. The secreted enterotoxins, including heat labile toxin (*LT*), heat-stable toxin type I (*STa*), heat-stable toxin type II (*STb*), and Shigella toxin type II (Stx2e) (2, 3), are the direct causes of diarrhea. They enhance the capillary permeability and increase the secretion of intestinal gland cells so that a large amount of liquid is accumulated in the small intestine, far exceeding the absorption capacity of the small intestine and leading to diarrhea. The loss of water, sodium, and carbonate ions results in dehydration, metabolic acidosis, and hyperkalemia, and it may induce heart failure and even death (4).

In order to prevent piglet diarrhea caused by ETEC, pregnant sows are usually immunized with live non-enterotoxigenic *E. coli* or protective antigens such as recombinant antigens of fimbriae and enterotoxins. Newborn piglets obtain passive protective antibodies through the colostrum (5, 6). Some breeds of sows cannot express receptors related to the adhesion of fimbriae, such as K88 receptor-negative sows, which do not produce or transfer specific antibodies against K88 in colostrum even post-immunization. These heterozygous piglets cannot obtain effective passive protection (1). The passive protection obtained through nursing does not effectively prevent post-weaning diarrhea. Egg yolk antibodies against adhesion fimbriae and enterotoxins might play a role in this period (7). In addition, some probiotics, such as *Bacillus subtilis, Bacillus licheniformis, Clostridium butyricum*, and yeasts, are administered to piglets. These probiotics use the nutrients in the intestinal tract for reproduction (8). During reproduction, lactic acid, butyric acid, antimicrobial peptides, and other substances are produced, causing the pH of the intestinal environment to decrease and inhibiting the growth of harmful bacteria. Probiotics interact with harmful bacteria such as *Escherichia coli, Salmonella*, and *Clostridium welchii* to compete for space and nutrition (9). Recently, a recombinant *Lactobacillus* expressing K88 and K99 fimbria was used to immunize mice orally. Among the immunized mice, 85% resisted a wild ETEC strain (10), indicating that this may be a useful new strategy in controlling piglet diarrhea.

The *LT* and *STb* genes are located in a large plasmid in ETEC K88ac, a dominant ETEC expressing K88 fimbria that is related to diarrhea (11, 12). *LT* plays a role in assisting bacterial adhesion and acts as a good adjuvant for mucosal immunity. Research has indicated that it might significantly enhance the responses of the humoral immune system and induce a high level of antibodies against the co-immune antigens in the intestines and serum, while at the same time avoiding immune tolerance and triggering a long-term memory effect to those co-immune antigens (13, 14). The subunit A of LT contains a domain with ADP-ribosylating activity. Subunit A can bind to NAD, and the serine of amino acid 63 of subunit A is located at the bottom of the NAD-binding chamber, which is closely related to virulence (15). Mutating serine (63) to lysine might cause the virulence of recombinant protein LT to be greatly reduced while retaining its strong adjuvanticity (16).

Our purpose was to construct an oral whole-cell vaccine against K88ac ETEC. In this paper, we developed an attenuated *E. coli* by mutating nucleotides 187–189 of *LT* from TCT (serine) to AAA (lysine) and deleting *STb* using λ-Red homologous recombination. This isolate could efficiently protect vaccinated mice against wild ETEC.

## Materials and Methods

### Animals, Bacterial Strains, Plasmids, and Primers

Eight-week-old female Balb/c mice and Japanese big-ear white rabbits with body weights between 2 and 3 kg were purchased from the Institute of Comparative Medicine, Yangzhou University (Jiangsu, China). Animals were raised under a standard 12-h light/dark cycle, in conventional housing conditions at 20 ± 1°C and 50 ± 10% relative humidity. All of the experimental animal operating procedures were in line with the guidelines for the management and use of experimental animals (2016 edition) and approved by the Animal Care and Use Committee of Yangzhou University. K88ac ETEC strain C83902 (*LT*^+^*, STb*^+^), used to construct the attenuated *E. coli* K88ac, was from the China Institute of Veterinary Drug Control (Beijing, China). DH5α competent cells and pUC18 plasmid were purchased from Takara Biotechnology (Dalian) Co., Ltd. Plasmids pSG76-CS and pSTKST as reported (17) were kindly provided by Prof. Lijun Zhang, Shenyang Agricultural University (Liaoning, China). Monoclonal antibody against a factor of K88 fimbriae was kindly provided by Prof. Song Gao, Yangzhou University. The primers (Table 1) used in this paper were designed according to the plasmid pUMNK88 (GenBank accession No. CP002730) and synthesized by Sangon Biotech (Shanghai) Co., Ltd., China.

**Table 1:** Primers used in constructing *E. coli* K88ac *LT(S63K)*Δ*STb*.

**Primer**	**Sequence (5^′^>**3′**)**	**Size (bp)**
P1	ctgaattcCGCTGCATTATTGATTTTAGGAC	378
P2	cgcccgggCACCTCAACCTTATTGCTATAAG	
P3	cgcccgggTATATTTATCAATAGCATTCAGCACC	356
P4	gcgaagctt CCGGATGATCTCTAAATATGAAT	
P5	CATATAAAAGCCCACTGG	1034
P6	CTTTTTATGAAAAATTATTTTTG	
P7	ATTGTTGACATGAACAGC	800
P8	GCGATCTCCTTCGTTAGG	
P9	gaggaacacaaaccggctttgtcagatatgatgacggatatgtttccactAAActtagtttgagaagtgctcac	1390
P10	cgagctcggtacccggggatccgcTAGGGATAACAGGGTAATgtcctgctaagtgagcacttctcaaactaagTTTag	
P11	gaatatcctgataatatagactgtcctgctaagtgagcacttctcaaactaagATTACCCTGTTATCCCTAtagcggccgcaaaaattaaaaatgaag	
P12	GAGGAACACAAACCGGCTTTG	106
P13	GAATATCCTGATAATATAGACTG	
P14	TAGAGACCGGTATTACAGAAATCTGA	282
P15	TCATCCCGAATTCTGTTATATATGTC	

### Construction of Mutated *E. coli* K88ac *LT(S63K)*Δ*STb* From C83902

**Figure 1:**
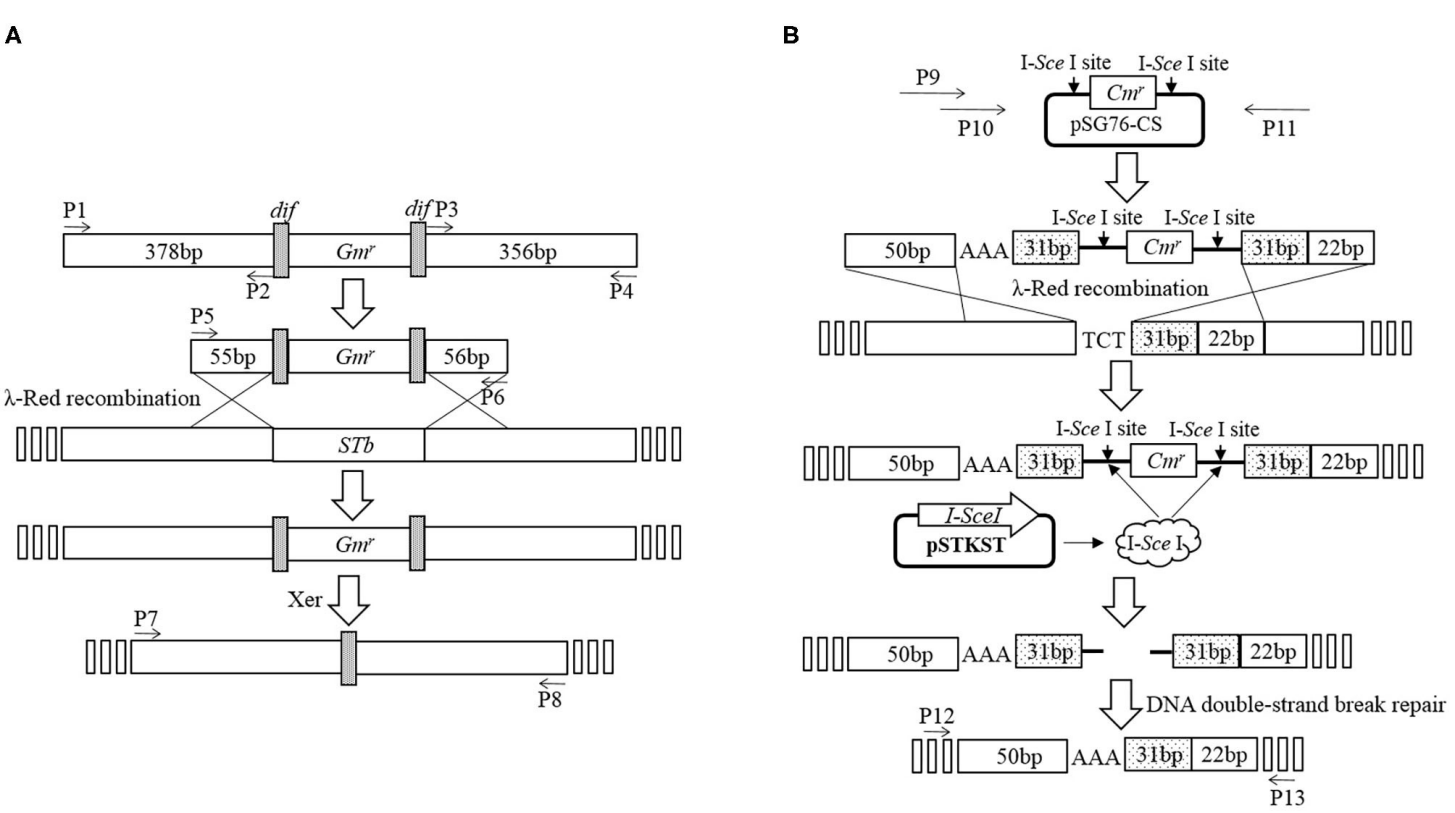
Construction of mutated *E. coli* K88ac *LT(S63K)*Δ*STb*. **(A)**. Deletion of *STb* to construct of *E. coli* K88ac ETECΔ*STb* by λ-Red homologous recombination; **(B)**. Construction of *E. coli* K88ac *LT(S63K)*Δ*STb* based on *E. coli* K88ac ETECΔ*STb* by mutating nucleotides 187–189 of *LT* from TCT (serine) to AAA (lysine) using λ-Red homologous recombination.

Deletion of *STb* was performed using an *E. coli* gene knockout kit (Jiangsu RuiYang BioTech Co., Ltd., China) as protocol and the construction process as shown in Figure 1A. Briefly, PCR fragments using primers P1/P2 and P3/P4 amplified from the C83902 strain were cloned into restriction endonuclease sites *Eco*R I/*Sma* I and *Sma* I/*Hin*d III of plasmid pUC18, then the *dif-Gm-dif* DNA digested with *Sma* I was inserted into the restriction endonuclease *Sma* I site. The constructed plasmid above was used as the template, and the recombinant cassette containing the left homologous arm, right homologous arm, and *dif-Gm-dif* amplified by PCR using primers P5/P6 was transformed into C83902 with plasmid pKD46 induced by L-arabinose at a final concentration of 4 μg/ml. After homologous recombination, the gentamicin resistance gene (*Gm*^*r*^) was deleted by the *dif/*Xer recombinant system of the *E. coli*, and a 34-bp sized *dif* sequence was left in the large plasmid of the *E. coli* named K88ac ETECΔ*STb*, which was verified by PCR using primers P7/P8.

Using plasmid pSG76-CS as template, another recombinant cassette containing left and right homologous arms, mutated nucleotides, and chloramphenicol resistance gene (*Cm*^*r*^) with two endonuclease I-*Sce*I sites on two sides was amplified by overlapping PCR using primers P9/P10/P11. The PCR product was transformed into K88ac ETECΔ*ST1* with plasmid pKD46 induced by L-arabinose to insert the amplicon into *LT* by λ-Red recombination. Then, plasmid pKD46 was removed at a temperature of 40°C and the plasmid pSTKST was transformed into the recombinant *E. coli*. After being induced by chlorotetracycline (Shanghai Yuanmu BioTech Co., Ltd., China) at a final concentration of 30 μg/ml, endonuclease I-*Sce*I expressed by plasmid pSTKST was used to delete the *Cm*^*r*^ gene. The large plasmid in *E. coli* was repaired by the method of broken-end recombination, and the mutation was fixed in *LT*. Then, plasmid pSTKST was removed at 40°C and the recombinant *E. coli* named K88ac *LT(S63K)*Δ*STb* was constructed and verified by sequencing the PCR product amplified using primers P12/P13 as Figure 1B.

### Indirect Immunofluorescence Assay

*E. coli* strains including K88ac *LT(S63K)*Δ*STb*, C83902, and DH5α were inoculated in TSB liquid medium and grown for 36 h. One ml of the bacterial solutions was centrifuged at 8,000 g/min for 5 min, washed gently with phosphate buffered saline (PBS) using a wide-bore tip, and resuspended in 1 ml PBS. Five μL of the suspensions was coated on glass slides and immobilized using the heat fixing method. The fixed bacteria were blocked with 5% goat serum for 15 min at room temperature and rinsed with PBS. Then, the slides were incubated with murine monoclonal antibody against a factor of K88 fimbriae diluted in 5% goat serum for 45 min. After being washed with PBS 3 times, the bacteria were incubated with 1:10,000 dilution of goat anti-mouse IgG conjugated with FITC (Abcam, UK) for another 45 min. The glass slides were washed in PBS 3 times, and the dyed bacteria were observed under the oil immersion lens of the microscope.

### Growth Characteristics

*E. coli* K88ac *LT(S63K)*Δ*STb* and C83902 were inoculated into liquid LB medium (pH = 7.2) and cultured overnight at 37°C. After the concentration was adjusted to OD600 = 0.04 with a UV spectrophotometer, the bacterial suspensions were transferred into LB medium at ratio of 1:100 and cultured at 37°C with shaking at 220 rpm. At the time points of 1.5 h, 3 h, 4 h, 6 h, 8 h, 10 h, 12 h, 14 h, and 16 h post-inoculation, the OD600 values of the suspensions were measured and the growth rate of the attenuated strain was compared with that of wild bacterium.

### *In vivo* Rabbit Ligated Ileal Loop Assay

*E. coli* bacteria including C83902, K88ac *LT(S63K)*Δ*STb*, and DH5α were grown in an LB medium with horizontal shaking at 37°C overnight, then transferred into a 10-ml CAYE medium at a ratio of 1:100 and cultured at 37°C. Seventy-two hours later, 2,640 units of polymyxin B were added and cultured for an extra 0.5 h. The bacterial suspensions were centrifuged at 10,000 × *g* for 10 min, and the obtained cell-free culture supernatants were filtered with sterile 0.22 μm-pore filters (Millipore, France).

The rabbit ligated ileal loop assay was performed as described with slight modifications (18). In detail, two Japanese big-ear white rabbits were fasted for 24 h and then anesthetized by the intramuscular injection of xylazine hydrochloride (SuMianXin; Tat Animal Pharmaceutical Co., Ltd. of Jilin Province, China) and zolazepam chloridrate (Zoletil; Virbac Sante Animal, France) at a dose of 0.08 ml/kg and 0.04 ml/kg, respectively. Using laparotomy, the empty mid-ileums were exposed. Ileal loops at lengths of 5 cm were ligated at intervals of 3 cm. Inoculums of 1 ml of cell-free culture supernatants or sterile CAYE medium as control were inoculated into each ligated loop of a rabbit using a 25-gauge needle, and repeated in another rabbit. After the abdominal cavities were closed, the rabbits were kept for 12 h, then euthanized by carbon dioxide inhalation. The volume of secretion in each ligated loop was measured, and the average ratio (the volume of liquid to the length of the intestine) of each sample was calculated. The average ratio was higher than 1.0, indicating that the enterotoxin existed in cell-free culture supernatant.

### Infection Study *in vivo*

The plasmid pUC18 was transformed into *E. coli* K88ac *LT(S63K)*Δ*STb* and C83902, and the two recombinant bacteria were reproduced in TSB medium with 1 μg/ml ampicillin. Forty-eight hours before the inoculation of bacteria, fructose and ampicillin were added to the animals' drinking water at concentrations of 67 μg/ml and 1 μg/ml respectively to encourage the mice to drink and get rid of the other bacteria; 12 h before inoculation, the mice were fasted; and 4 h before inoculation, the drinking water was changed into sterile water and all of mice were injected with cimetidine (50 mg/kg) intraperitoneally. The mice were divided into three groups and raised in independent units. According to the preliminary experimental results, each mouse of group 1 (*n* = 10), group 2 (*n* = 10), and group 3 (*n* = 10) was inoculated 0.4 ml (approximately 10^9^ CFU) of K88ac ETEC (C83902), K88ac *LT(S63K)*Δ*STb*, and sterile PBS by intragastric administration, respectively. Clinical symptoms were observed in the following 7 days, and clinical signs were recorded and scored every day as described with slight modifications (19): 0, healthy; 1, mental depression and ruffled fur; 2, hunchbacked appearance, shivering, and dark urine; 3, mild diarrhea; 4, severe diarrhea; 5, moribund or dead. The feces were collected over the following 33 days for detecting the inoculated *E. coli* and IgA secreted in the intestinal tract.

### *E. coli* K88ac *LT(S63K)ΔSTb* and Wild Strain Detection

Feces collected were inoculated into the TSB liquid medium with 1 μg/ml ampicillin and shaken in an orbital incubator at a speed of 220 rpm for 16 h. PCR assay was performed to detect *LT* or mutant *LT* genes in wild or attenuated strains using primers P14 and P15 with an initial denaturation at 94°C for 5 min, followed by 30 cycles of denaturation at 94°C for 30 s, annealing at 54°C for 30 s, extension at 72°C for 1 min, and an extra extension at 72°C for 5 min. PCR products were visualized in 1.5% agarose gel stained with ethidium bromide using the gel image documentation system.

### IgA Detection by Indirect ELISA

The K88ac fimbriae of C83902 were isolated and purified as described (6); the purified fimbriae were adjusted to a concentration of 10 ng/ml in 0.05 mol/l carbonate buffered saline at a pH of 9.6. Then, 100 μl of the diluted antigen was coated into the wells of the 96-well assay plates (Corning, America) overnight at 4°C. The plates were washed twice with PBS containing 0.05% Tween 20 (PBST) and blocked with 200 μl/well of PBST with 5% non-fat dry milk for 1 h at 37°C. The plates were then washed twice more with PBST. Subsequently, 0.5 ml PBS was added into 0.1 g mice feces collected at intervals of 3 days, mixed completely, and centrifuged at 12,000 rpm for 5 min. The supernatants were collected and transferred into 96-well plates at 100 μl/well, incubated for 1 h at 37°C, and washed three times with PBST. A 1:5,000 dilution of HRP-conjugated goat anti-mouse IgA antibody (Abcam, UK) in a blocking solution was dispensed into the plates at 100 μl/well and incubated for 1 h at 37°C. The plates were washed again with PBST, then 100 μl/well of 3,3,5,5-tetramethylbenzidine substrate (Sangon Biotech, China) was added, and the reaction was stopped with 50 μl/well of 2 mol/L sulfuric acid solution 15 min later. The antibody level was measured by the microplate reader (Biotech, America) at a wavelength of 450 nm.

### Protection Assay

Fifty mice were divided into three groups (five mice each in Group 1 and Group 2 as control groups and 40 mice in Group 3 as the experimental group), and mice in all of the groups were treated with fructose, antibiotic, and cimetidine as described above. Then, the mice in Group 1 took C83902 with plasmid pUC18 orally at a dose of 10^9^ CFU/each, and mice in Group 2 and Group 3 took *E. coli* K88ac *LT(S63K)*Δ*STb* with plasmid pUC18 orally at a dose of 10^9^ CFU/each. Group 3 performed as the challenge group. Five mice in Group 3 were selected at random and raised in independent units, then treated with C83902 harboring plasmid pUC18 at a dose of 10^9^ CFU/each at intervals of 24 h post-inoculation over the following 7 days. The last five mice were treated on the 24th day. Clinical signs were recorded over the 3 days following the treatment and scored as described above.

### Analysis

Data were analyzed using the GraphPad Prism 5.00 software.

## Results

### Generation of *E. coli* K88ac *LT(S63K)ΔSTb*

**Figure 2:**
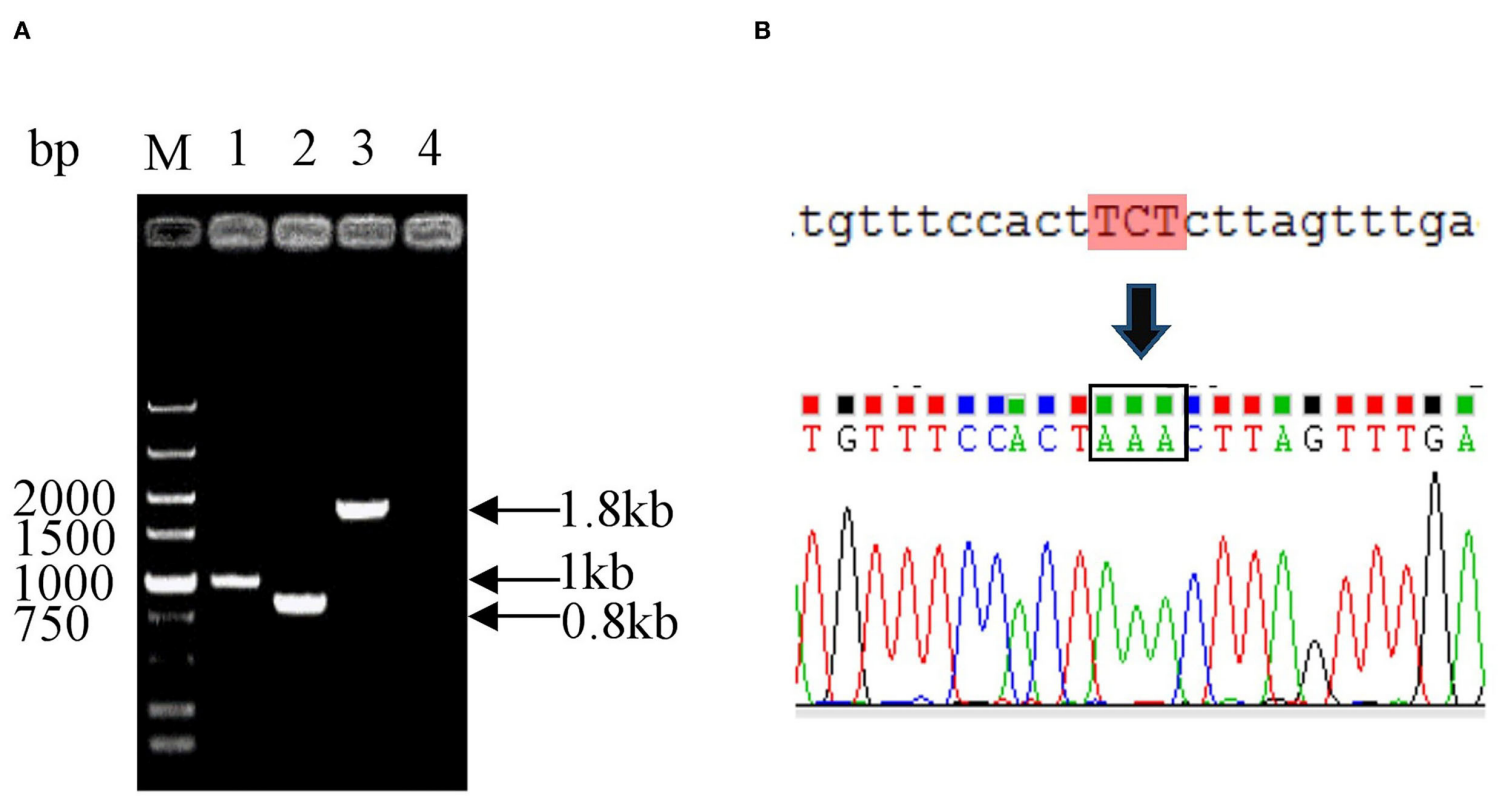
Identification of mutated *E. coli* K88ac *LT(S63K)*Δ*STb*. **(A)**. Identification of *E. coli* K88ac ETECΔ*STb* by PCR, M. DNA marker, 1. Wild strain C83902, 2. *E. coli* K88ac ETECΔ*STb*, 3. *E. coli* K88ac ETECΔ*STb* before removal of gentamicin resistance gene, 4. Negative control; **(B)**. Sequence result of mutating TCT (serine) to AAA (lysine).

To generate the mutant *E. coli* K88ac, two λ-Red recombinations were carried out. Firstly, the λ-Red recombination system was used to knock out the *STb* gene located on the large plasmid harbored in C83902. The constructed K88ac ETECΔ*STb* was verified by PCR and sequence analysis, and the results indicated that the *STb* gene was replaced by a 34-bp-sized *dif* (Figure 2A). Secondly, based on the procedure above, a markerless mutation was performed to change the serine at position amino acid 63 of heat-labile toxin into lysine using λ-Red recombination again. Sequencing results showed that the codon TCT(S) was mutated to a codon AAA(K) (Figure 2B), and *E. coli* K88ac *LT(S63K)*Δ*STb* was obtained.

### K88ac *LT(S63K)ΔSTb* Could Efficiently Express Fimbriae

**Figure 3:**
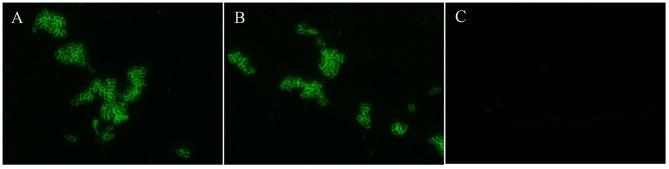
Identification of K88ac expressed on the surface of bacteria by IFA. **(A)**. C83902; **(B)**. *E. coli* K88ac *LT(S63K)*Δ*STb*; **(C)**. *E. coli* DH5α as negative control.

Usually, fimbriae of K88ac expressed on the surface of *E. coli* is encoded by the genes in the large plasmid, which is important in the adhesion of susceptible cells in intestinal mucosa and plays important roles in the pathogenic process. To avoid knocking out fimbriae genes by mistake, K88ac were detected by immunofluorescence assay. The results of IFA showed that there was bright fluorescence around the surfaces of K88ac *LT(S63K)*Δ*STb* and C83902, but no fluorescence on the surface of control DH5α as shown in Figure 3, indicating that the mutation and knockout of enterotoxin genes had no effect on the expression of K88ac fimbriae.

### K88ac *LT(S63K)ΔSTb* Showed Similar Growth Kinetics to the Wild Strain

**Figure 4:**
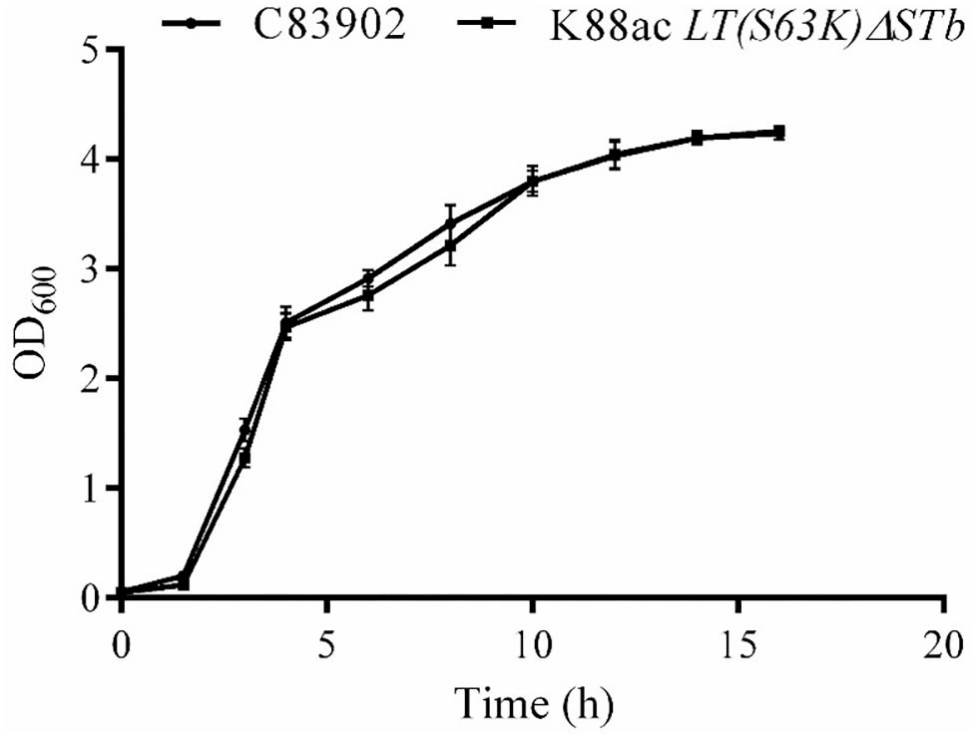
Growth kinetics of K88ac *LT(S63K)*Δ*STb* compared with the wild strain. The growth rate of K88ac *LT(S63K)*Δ*STb* was a little lower in the first several hours, but the bacterial reproduction rate of K88ac *LT(S63K)*Δ*STb* reached the same level as C83902 10 h later.

To ensure the growth character of *E. coli* K88ac *LT(S63K)*Δ*STb*, the fresh bacterial fluid of the attenuated *Escherichia coli* was inoculated into the LB medium at a ratio of 1:100 and cultured at 37°C. C83902 was set as the control, and concentrations of bacterial fluids at different times post-inoculation were measured using the OD_600_ value obtained by a UV spectrophotometer. As shown in Figure 4, the growth rate of the attenuated strain was a little lower compared with that of the original bacterium in first several hours, but at 10–16 h post-inoculation the bacterial reproduction quantity of the two bacteria reached the same level. This revealed that the attenuated strain had little effect on bacterial reproduction.

### K88ac *LT(S63K)ΔSTb* Could Not Efficiently Secrete the Active Enterotoxin

**Figure 5:**
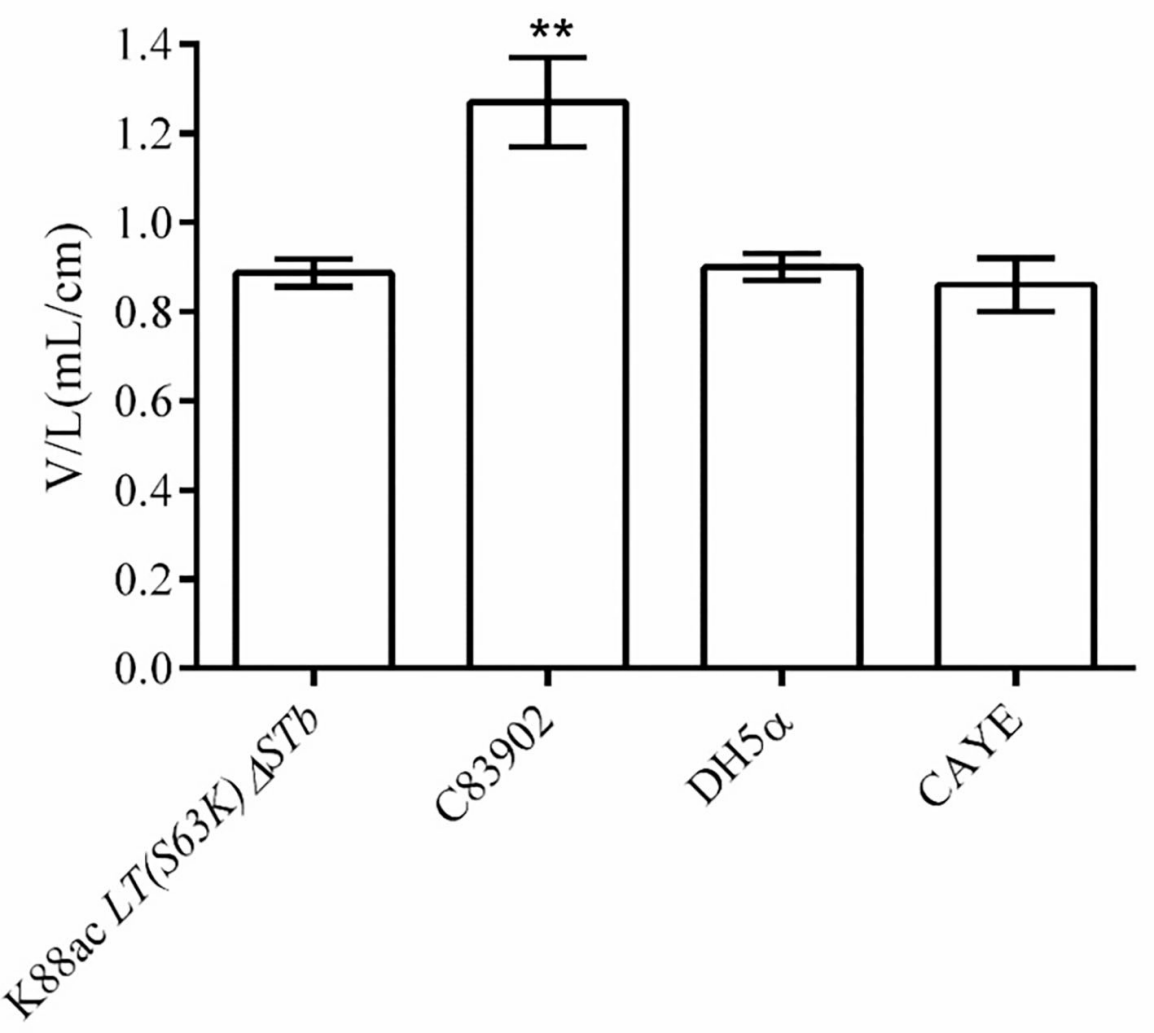
Detection of the active enterotoxin by rabbit ligated ileal loop assay. The average ratio (the volume to the corresponding ligated loop length, V/L) of inoculating the culture supernatant of C83902 was 1.27, but that of inoculating culture supernatants of *E. coli* K88ac *LT(S63K)*Δ*STb*, DH5α, and sterile CAYE medium were no more than 0.9 (^**^p < 0.01). Asterisks indicate very significant differences between groups.

To detect the enterotoxin secretion of the mutated strain *E. coli* K88ac *LT(S63K)*Δ*STb*, the enterotoxins were detected using rabbit ligated ileal loop assay. Sterile culture supernatant of K88ac *LT(S63K)*Δ*STb* was injected into rabbit ligated ileal loops. At the same time, the controls of the sterile culture supernatant of *E. coli DH5*α, C83902, and CAYE medium were also injected into different rabbit ligated ileal loops separately. Twelve hours later, the volumes of collected intestinal liquid in each ligated loop were measured and the ratio of fluid volume to the corresponding ligated loop length was calculated. The results indicated that the average ratio of positive control inoculating culture supernatant of K88ac ETEC (C83902) was 1.27, but others were no more than 0.9 as shown in Figure 5, demonstrating that the mutated strain could not effectively secrete active diarrhea-causing enterotoxin.

### K88ac *LT(S63K)ΔSTb* Was Highly Attenuated in Mouse Models and Caused Mucosal Immune Response

Mice of Group 1 and Group 2 were inoculated with C83902 and K88ac *LT(S63K)*Δ*STb* at doses of 10^9+^CFU/each, and mice of Group 3 were inoculated with sterile PBS as a control. The clinical scores of each group are recorded in Table 2. In detail, 6 h later, the clinical symptoms of the mice in Group 1 were as follows: mental depression, slow reactions, ruffled fur and hunchbacked appearances, shivering, dark urine, and diarrhea. Six mice died 12–24 h post-inoculation, and the remaining mice gradually recovered over the following days. The survival rate of mice in Group 1 was 40%. Except for mild and transient diarrhea seen in one individual mouse of Group 2, the rest of Group 2 and control Group 3 looked quite normal in subsequent experiments. The survival rates of mice in Group 2 and Group 3 were 100%. After dissecting the dead mice of Group 1, inflation, emptiness, and yellow effusion in the intestinal tracts were found. The mouse with mild diarrhea in Group 2 was killed by cervical dislocation, and a small amount of fluid was found in the intestinal tract. The intestinal tract of the control mouse in Group 3 was normal, indicating that the virulence of *E. coli* K88ac *LT(S63K)*Δ*STb* to the mouse model was greatly reduced.

**Table 2:** Clinical score recorded after inoculation of *E. coli* C83902 and K88ac *LT(S63K)*Δ*STb*.

**DPI**	**C83092**	**K88ac *LT(S63K)STb***	**Control**
	**(Group 1)**	**(Group 2)**	**(Group 3)**
1	44	3	0
2	56	3	0
3	62	3	0
4	62	3	0
5	62	3	0
6	62	3	0
7	62	3	0

*There were 30 mice that were equally divided into 3 groups and raised in independent units. Mice of each group were inoculated with C83902, K88ac LT(S63K)ΔSTb, and sterile PBS as control by intragastric administration at a dose of approximately 10^9^ CFU/each. Clinical symptoms were observed over the following 7 days. Clinical signs were recorded and scored. Clinical symptoms of the mice in Group 1 were exhibited at the 6th hour post-inoculation: 6 mice died, 2 mice had severe diarrhea, and the remaining 2 had mild diarrhea at the end of the first day post-inoculation (DPI); 4 mice had mild diarrhea on the second day, 2 mice were depressed with ruffled fur, and the remaining 2 mice appeared hunchbacked and moved slowly on the third day, then recovered on the following day. Except for mild and transient diarrhea in a single mouse on the first day post-inoculation with K88ac LT(S63K)ΔSTb in Group 2, the mice in Group 2 and control Group 3 looked quite normal during the experimental period*.

**Figure 6:**
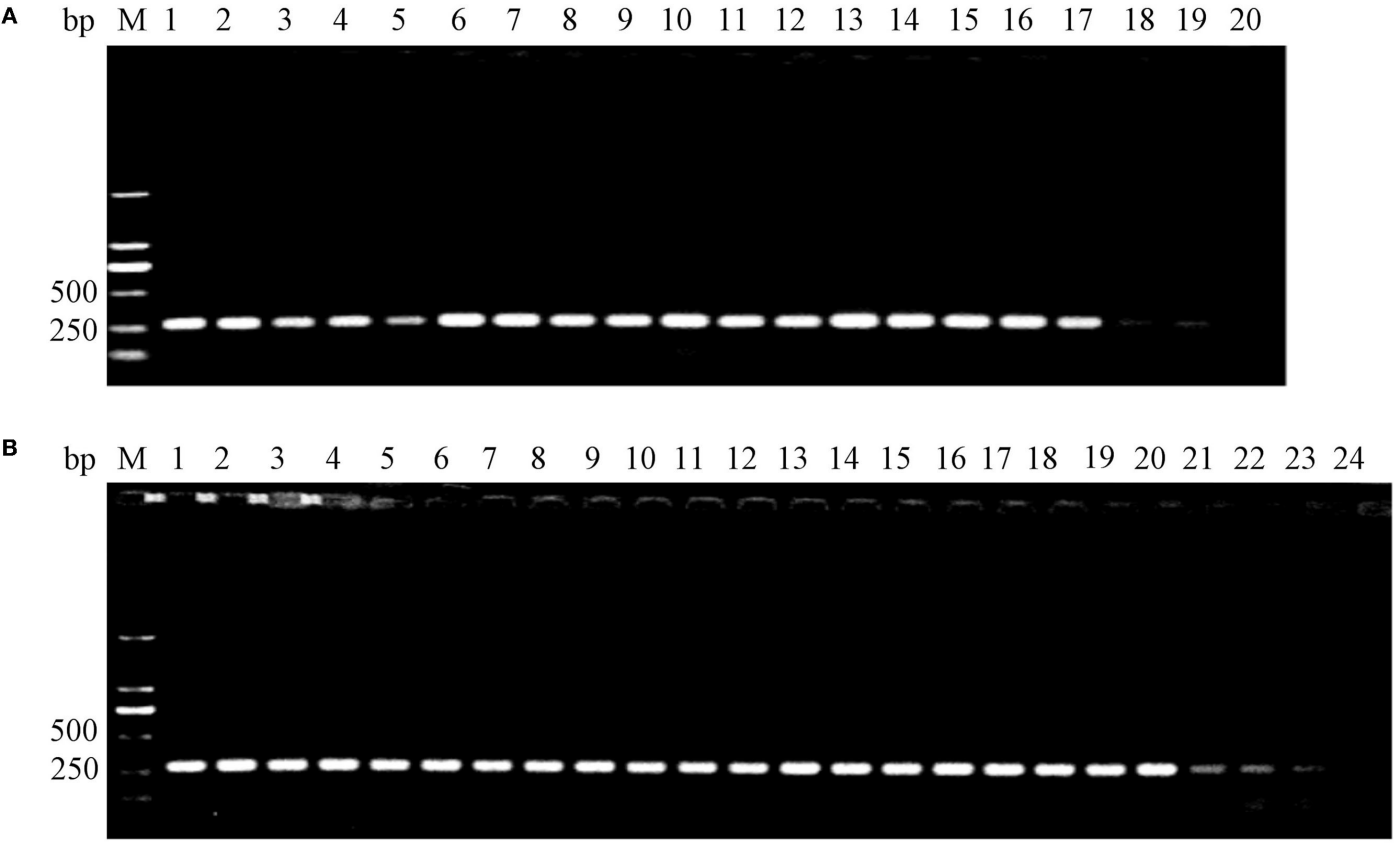
Detection of surviving *E. coli* K88ac *LT(S63K)*Δ*STb* and C83902 in mouse intestines by PCR. **(A)**. C83902 in feces could survive for 19 days; **(B)**. *E. coli* K88ac *LT(S63K)*Δ*STb* in feces could survive for 23 days.

Feces were collected separately from the three groups every day. After the enrichment of bacteria, the *LT* and mutant *LT* genes in the wild or attenuated strains were detected by PCR to ensure the duration in mouse model. Results indicated that the wild strain could persist for 19 days in mouse intestines, while the attenuated strain could persist for 23 days as shown in Figure 6.

**Figure 7:**
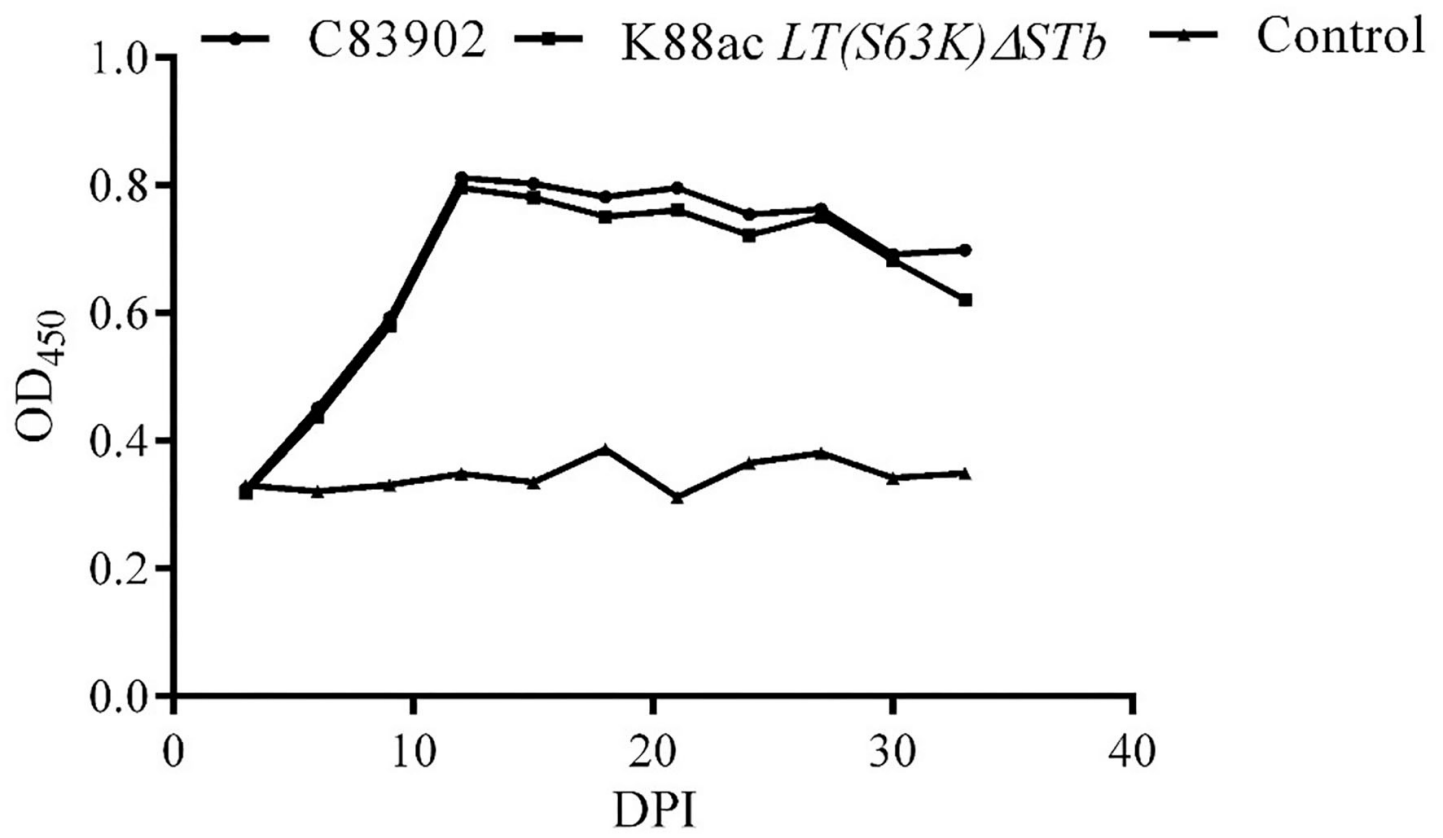
Detection of IgA against K88ac in feces by indirect ELISA. IgA against K88ac could be detected at the earliest on the 6th day post-inoculation (DPI), the highest titer of IgA was found on the 12th day, and high titers of IgA anti-K88ac could still be detected at the 33th day.

IgA against fimbriae K88ac in feces was detected by indirect ELISA. From the results of indirect ELISA shown in Figure 7, IgA could be detected out as early as the 6th day after inoculation. As time went on, the highest titer of IgA was found on the 12th day, and a high titer of IgA anti-K88ac could still be detected at the 33rd day. This indicated that the attenuated strain could cause a mucosal immune response in the mouse intestinal tract.

### K88ac *LT(S63K)ΔSTb* Provided Efficient Protection Against the Lethal Challenge

Mice of Group 1 (*n* = 5), Group 2 (*n* = 5), and Group 3 (*n* = 40) were treated with fructose, antibiotic, and cimetidine. Then, the mice in Group 1 took C83902 with plasmid pUC18 orally at a dose of 10^9^ CFU/each while the mice in Group 2 and Group 3 took *E. coli* K88ac *LT(S63K)*Δ*STb* with plasmid pUC18 orally at a dose of 10^9^ CFU/each. Five mice in Group 3 were randomly selected and moved into a new unit at an interval of 24 hours and treated with C83902 containing plasmid pUC18 at a dose of 10^9^ CFU/each until 168 h post-inoculation with the attenuated bacterium. The last five mice were treated at the 24 h post-inoculation, when the attenuated bacteria were eliminated from the mouse intestines. Clinical presentations were scored in the following 72 h post-treatment, and the results indicated that the mice in Group 1 exhibited mental depression, slow reactions, ruffled fur, hunchbacked appearances, and shivering. Four mice died 12–24 h post-inoculation, and one mouse survived at 72 h post-inoculation. The mice in Group 2 looked normal in appearance and behavior. In Group 3, all the mice challenged with the original bacterium were normal until the 5th challenge; one mouse exhibited a hunched back and slow reactions in the following 24 h and recovered the next day at the 6th challenge; one mouse in each unit exhibited mild diarrhea at 24 h, then recovered quickly in the 7th and 8th challenge. The clinical scores were recorded in Table 3, and the survival rates of mice in Group 1, Group 2, and Group 3 were 20, 100, and 100%, respectively.

**Table 3:** Clinical score recorded after inoculation with *E. coli* C83902, K88ac *LT(S63K)*Δ*STb*, and being challenged with C83902.

**DPI**	**C83092 (Group 1)**	**K88ac *LT(S63K)STb* (Group 2)**	**K88ac** ***LT(S63K)STb*** **(Group 3)**							
			**Challenge 1**	**Challenge 2**	**Challenge 3**	**Challenge 4**	**Challenge 5**	**Challenge 6**	**Challenge 7**	**Challenge 8**
1	23	0		-	-	-	-	-	-	-
2	25	0	0	-	-	-	-	-	-	-
3	26	0	0	0	-	-	-	-	-	-
4	-	-	0	0	0	-	-	-	-	-
5	-	-	-	0	0	0	-	-	-	-
6	-	-	-	-	0	0	0	-	-	-
7	-	-	-	-	-	0	0	2	-	-
8	-	-	-	-	-	-	0	2	3	-
9	-	-	-	-	-	-	-	2	3	-
10	-	-	-	-	-	-	-	-	3	-
24										3
25										3
26										3

“-” means no recording took place.

*Fifty mice were divided into Group 1 (n = 5), Group 2 (n = 5), and Group 3 (n = 40). The mice in Group 1 took C83902 orally at a dose of 10^9^ CFU/each, and the mice in Group 2 and Group 3 took E. coli K88ac LT(S63K)ΔSTb orally at doses of 10^9^ CFU/each. Five mice in Group 3 were randomly removed into a new unit at an interval of 24 h and challenged by C83902 at a dose of 10^9^ CFU/each until 168 h post inoculating attenuated bacterium, and the last 5 mice were challenged at the 24th day when the attenuated bacteria were eliminated from the intestinal tract. Clinical presentations were scored in the following 72 h post-challenge. The results indicated that the mice in Group 1 exhibited mental depression, slow reactions, ruffled fur, hunchbacked appearances, and shivering in the following hours. Four mice died and 1 mouse survived with mild diarrhea at the end of first day post-inoculation. The mouse appeared hunchbacked and moved slowly at the end of the second day and exhibited light depression at the end of third day post-inoculation. The mice in Group 2 looked normal in appearance and behavior. In Group 3, all the mice challenged with the wild strain were quite normal until the 5th challenge; after the 6th challenge one mouse was hunchbacked and had slow reactions at the end of first day post-challenge and recovered the next day; in the 7th and 8th challenge, one mouse in each unit exhibited mild diarrhea at the end of the first day post challenge, then recovered the next day*.

## Discussion

As described previously, our aim was to construct an attenuated strain for an oral whole-cell vaccine against K88ac ETEC based on mutating nucleotides 187–189 of *LT* from TCT (serine) to AAA (lysine) and deleting *STb*. λ-Red homologous recombination is a common technique used in the mutation and knockout of genes located in chromosomes, but the genes of *LT* and *STb* were in free large plasmids instead of bacterial chromosomes. The unknown copy number of the plasmid, which might lead to incomplete knockout of genes, was the biggest obstacle to using homologous recombination. In this paper, we realized the traceless point mutation in *LT* and deletion of *STb* using two λ-Red recombination systems and constructed the *E. coli* K88ac *LT(S63K)*Δ*STb* from wild paternal *E. coli* C83902 (*K88ac*^+^, *LT*^+^, *STb*^+^), which was verified by PCR and DNA sequencing. The following enterotoxin detection by rabbit ligated ileal loop assay and *in vivo* test in mice proved that the constructed strain was an attenuated *E. coli*.

In the process of λ-Red homologous recombination, imperceptible mistaken deletions may lead to changes in the biological characteristics of bacteria. The characteristics of the mutated bacterium, such as expression of adhesive fimbriae and speed of proliferation, were compared with the original strain. The results demonstrated that the attenuated bacteria expressed the K88ac fimbriae normally, and there was no significant difference in the proliferation rate compared to the wild strain C83902, which provided a reliable theoretical basis for further study of the attenuated strain in animals.

Usually mice are used as animal models in studying pathogenicity and oral ETEC vaccines (9, 20–22). In this paper, the Balb/c mice were inoculated orally with attenuated strains and the pathogenicity of the attenuated strain was significantly decreased compared with the wild parental strain C83902. After taking the attenuated strain, the mice could effectively resist wild K88ac ETEC at doses of 10^9^ bacteria in the following 7 days. Usually the first 7 days is very critical for newborn piglets, when K88ac ETEC may result in yellow scour.

IgA is the predominant antibody at the mucosal surface (23). IgA against fimbrial proteins can neutralize the corresponding antigen and inhibit the adhesion of ETEC strains harboring specific receptors present on the brush borders of villous enterocytes (24). The dates showed that the IgA against K88ac secreted by small intestinal mucosa in mice could be detected on the 6th day after inoculation by indirect ELISA. The titer of IgA then gradually increased as time passed and reached the peak at the 12th day, which indicated that the attenuated strain could stimulate mucosal immune response and secrete antibodies against bacteria. However, there was a 5-day blank window period of antibody formation after immunization. Ecological agents such as probiotics can promote the colonization of normal microbiota, increase digestion capacity, reduce intestinal pH value, and improve mucosal immunity (9). Another important role played by ecological agents is the priority occupying effect, which prevents adhesion and colonization by pathogenic bacteria such as *Yersinia enterocolitica* and ETEC (25). During the blank window period of IgA formation, the attenuated strain could protect mice against the wild strain C83902, which indicated it might act as a probiotic in the early stage after vaccination. The survival time of the attenuated strain reached 23 days in the intestinal tract of mice, and the results of a challenge at the 24th day post-inoculation revealed that the attenuated isolate as a vaccine could effectively protect the mice against wild ETEC. In addition, the attenuated isolate stayed in the intestines for a long time, it might have potential in the development of mucosal immune transport carrier.

In conclusion, this was the first demonstration of the generation of a high attenuated *E. coli* K88ac *LT(S63K)*Δ*STb* deletion. *Escherichia coli* K88ac *LT(S63K)*Δ*STb* could elicit mucosal immune response and provide efficient protection against the lethal challenge of ETEC in mouse models, highlighting the fact that *Escherichia coli* K88ac *LT(S63K)*Δ*STb* can be used as a live-attenuated vaccine candidate against ETEC. However, the attenuated phenotype and the protective efficacy of *Escherichia coli* K88ac *LT(S63K)*Δ*STb* in piglets need to be further evaluated.

## Data Availability Statement

The original contributions presented in the study are included in the article/supplementary materials, further inquiries can be directed to the corresponding author/s.

## Ethics Statement

The animal study was reviewed and approved by Animal Care and Use Committee of Yangzhou University.

## Author Contributions

XZ and SY completed most of the work. YY helped with rabbit surgery. DC, YF, HS, and JD gave some beneficial advice. FW designed most of the study. All authors contributed to the article and approved the submitted version.

## Conflict of Interest

The authors declare that the research was conducted in the absence of any commercial or financial relationships that could be construed as a potential conflict of interest. The reviewer GZ declared a shared affiliation with several of the authors, XZ, SY, DC, YY, HS, to the handling editor at time of review.

## References

[1] Fairbrother JM Nadeau E Gyles CL. Escherichia coli in postweaning diarrhea in pigs: an update on bacterial types, pathogenesis, prevention strategies. Anim Health Res Rev. (2005) 6:17–39. 10.1079/ahr2005105 16164007

[2] Dubreuil JD Isaacson RE Schifferli DM. Animal enterotoxigenic *Escherichia coli*. EcoSal Plus. (2016) 7:1–47. 10.1128/ecosalplus.ESP-0006-2016 PMC5123703 27735786

[3] Zhang W Zhao M Ruesch L Omot A Francis D. Prevalence of virulence genes in Escherichia coli strains recently isolated from young pigs with diarrhea in the US. Vet Microbiol. (2007) 123:145–52. 10.1016/j.vetmic.2007.02.018 17368762

[4] Gill DM Clements JD Robertson DC Finkelstein RA. Subunit number and arrangement in Escherichia coli heat-labile enterotoxin. Infect Immun. (1981) 33:677–82. 10.1128/IAI.33.3.677-682.1981 7026442 PMC350761

[5] Calinescu C Mulhbacher J Nadeau E Fairbrother JM Mateescu MA. Carboxymethyl high amylose starch (CM-HAS) as excipient for Escherichia coli oral formulations. Eur J Pharm Biopharm. (2005) 60:53–60. 10.1016/j.ejpb.2004.12.006 15848056

[6] Van den Broeck W Cox E Goddeeris BM. Induction of immune responses in pigs following oral administration of purified F4 fimbriae. Vaccine. (1999) 17:2020–9. 10.1016/s0264-410x(98)00406-x 10217602

[7] Owusu-Asiedu A Baidoo SK Nyachoti CM Marquardt RR. Response of early-weaned pigs to spray-dried porcine or animal plasma-based diets supplemented with egg-yolk antibodies against enterotoxigenic Escherichia coli. J Anim Sci. (2002) 80:2895–903. 10.2527/2002.80112895x 12462257

[8] Hill C Guarner F Reid G Gibson GR Merenstein DJ Pot B. Expert consensus document. The International Scientific Association for Probiotics and Prebiotics consensus statement on the scope and appropriate use of the term probiotic. Nat Rev Gastroenterol Hepatol. (2014) 11:506–14. 10.1038/nrgastro.2014.66 24912386

[9] Uyeno Y Shigemori S Shimosato T. Effect of probiotics/prebiotics on cattle health and productivity. Microbes Environ. (2015) 30:126–32. 10.1264/jsme2.ME14176 26004794 PMC4462921

[10] Wen LJ Hou XL Wang GH Yu LY Wei XM Liu JK. Immunization with recombinant Lactobacillus casei strains producing K99, K88 fimbrial protein protects mice against enterotoxigenic Escherichia coli. Vaccine. (2012) 30:3339–49. 10.1016/j.vaccine.2011.08.036 21856357

[11] Choi C Chae C. Genotypic prevalence of F4 variants (ab, ac, and ad) in Escherichia coli isolated from diarrheic piglets in Korea. Vet Microbiol. (1999) 67:307–10. 10.1016/s0378-1135(99)00046-2 10466506

[12] Ochi S Shimizu T Ohtani K Ichinose Y Arimitsu H Tsukamoto K. Nucleotide sequence analysis of the enterotoxigenic Escherichia coli Ent plasmid. DNA Res. (2009) 16:299–309. 10.1093/dnares/dsp015 19767599 PMC2762410

[13] Akker FVD Pizza M Rappuoli R Hol WGJ. Crystal structure of a non-toxic mutant of heat-labile enterotoxin, which is a potent mucosal adjuvant. Protein Sci. (2010) 6:2650–4. 10.1002/pro.5560061220 9416617 PMC2143616

[14] Horstman AL Bauman SJ Kuehn MJ. Lipopolysaccharide 3-deoxy-D-manno-octulosonic acid (Kdo) core determines bacterial association of secreted toxins. J Biol Chem. (2004) 279:8070–5. 10.1074/jbc.M308633200 14660669 PMC3525363

[15] O'Neal CJ Jobling MG Holmes RK Hol WG. Structural basis for the activation of cholera toxin by human ARF6-GTP. Science. (2005) 309:1093–6. 10.1126/science.1113398 16099990

[16] Pizza M Domenighini M Hol W Giannelli V Fontana MR Giuliani MM. Probing the structure-activity relationship of Escherichia coli LT-A by site-directed mutagenesis. Mol Microbiol. (1994) 14:51–60. 10.1111/j.1365-2958.1994.tb01266.x 7830560

[17] Hu FX Ding R Cui ZH Yu JL Liu LF Ao YH. Approaches and strategies of gene scarless knockout in the Escherichia coli genome. Letters Biotechnol. (2013) 24:552–7. 10.3969/j.issn.1009-0002.2013.04.026

[18] Syngkon A Elluri S Koley H Rompikuntal PK Saha DR Chakrabarti MK. Studies on a novel serine protease of a DeltahapADeltaprtV Vibrio cholerae O1 strain and its role in hemorrhagic response in the rabbit ileal loop model. PLoS ONE. (2010) 5:e13122. 10.1371/journal.pone.0013122 20927349 PMC2948034

[19] Kolpe AB Kiener TK Grotenbreg GM Kwang J. Display of enterovirus 71 VP1 on baculovirus as a type II transmembrane protein elicits protective B and T cell responses in immunized mice. Virus Res. (2012) 168:64–72. 10.1016/j.virusres.2012.06.014 22728446

[20] Laird RM Ma Z Dorabawila N Pequegnat B Omari E Liu Y. Evaluation of a conjugate vaccine platform against enterotoxigenic Escherichia coli (ETEC), Campylobacter jejuni and Shigella. Vaccine. (2018) 36:6695–702. 10.1016/j.vaccine.2018.09.052 30269917 PMC6223012

[21] Ran X Chen X Wang S Chang C Wen X Zhai J. Preparation of porcine enterotoxigenic Escherichia coli (ETEC) ghosts and immunogenic analysis in a mouse model. Microb Pathog. (2019) 126:224–30. 10.1016/j.micpath.2018.11.015 30428380

[22] Rodea GE Montiel-Infante FX Cruz-Cordova A Saldana-Ahuactzi Z Ochoa SA Espinosa-Mazariego K. Tracking bioluminescent ETEC during in vivo BALB/c mouse colonization. Front Cell Infect Microbiol. (2017) 7:187. 10.3389/fcimb.2017.00187 28560186 PMC5432549

[23] Virdi V Coddens A De Buck S Millet S Goddeeris BM Cox E. Orally fed seeds producing designer IgAs protect weaned piglets against enterotoxigenic Escherichia coli infection. Proc Natl Acad Sci USA. (2013) 110:11809–14. 10.1073/pnas.1301975110 23801763 PMC3718133

[24] de Oliveira IR Bessler HC Bao SN Lima RD Giugliano LG. Inhibition of enterotoxigenic Escherichia coli (ETEC) adhesion to Caco-2 cells by human milk and its immunoglobulin and non-immunoglobulin fractions. Braz J Microbiol. (2007) 38:86–92. 10.1590/S1517-83822007000100018

[25] Yu X Avall-Jaaskelainen S Koort J Lindholm A Rintahaka J von Ossowski I. A comparative characterization of different host-sourced lactobacillus ruminis strains and their adhesive, inhibitory, immunomodulating functions. Front Microbiol. (2017) 8:657. 10.3389/fmicb.2017.00657 28450859 PMC5390032

